# Genomic patterns linked to gray matter alterations underlying working memory deficits in adults and adolescents with attention-deficit/hyperactivity disorder

**DOI:** 10.1038/s41398-023-02349-x

**Published:** 2023-02-11

**Authors:** Kuaikuai Duan, Jiayu Chen, Vince D. Calhoun, Wenhao Jiang, Kelly Rootes-Murdy, Gido Schoenmacker, Rogers F. Silva, Barbara Franke, Jan K. Buitelaar, Martine Hoogman, Jaap Oosterlaan, Pieter J. Hoekstra, Dirk Heslenfeld, Catharina A. Hartman, Emma Sprooten, Alejandro Arias-Vasquez, Jessica A. Turner, Jingyu Liu

**Affiliations:** 1 Department of Electrical and Computer Engineering, Georgia Institute of Technology, Atlanta, GA USA; 2 Tri-Institutional Center for Translational Research in Neuroimaging and Data Science (TReNDS), Atlanta, GA USA; 3 Department of Computer Science, Georgia State University, Atlanta, GA USA; 4 Department of Psychology, Georgia State University, Atlanta, GA USA; 5 Department of Human Genetics, Donders Institute for Brain, Cognition and Behavior, Radboud University Medical Center, Nijmegen, The Netherlands; 6 Department of Psychiatry, Donders Institute for Brain, Cognition and Behavior, Radboud University Medical Center, Nijmegen, The Netherlands; 7 Department of Cognitive Neuroscience, Donders Institute for Brain, Cognition and Behavior, Radboud University Medical Center, Nijmegen, The Netherlands; 8 Department of Clinical Neuropsychology, Vrije Universiteit Amsterdam, Amsterdam, The Netherlands; 9 Department of Child and Adolescent Psychiatry, University Medical Center Groningen, University of Groningen, Groningen, The Netherlands; 10 Faculty of Behavioral and Movement Sciences, Vrije Universiteit Amsterdam, Amsterdam, The Netherlands; 11 Department of Psychiatry, University of Groningen, Groningen, The Netherlands

**Keywords:** Clinical genetics, ADHD

## Abstract

Attention-deficit/hyperactivity disorder (ADHD) is a highly heritable neurodevelopmental disorder, with onset in childhood and a considerable likelihood to persist into adulthood. Our previous work has identified that across adults and adolescents with ADHD, gray matter volume (GMV) alteration in the frontal cortex was consistently associated with working memory underperformance, and GMV alteration in the cerebellum was associated with inattention. Recent knowledge regarding ADHD genetic risk loci makes it feasible to investigate genomic factors underlying these persistent GMV alterations, potentially illuminating the pathology of ADHD persistence. Based on this, we applied a sparsity-constrained multivariate data fusion approach, sparse parallel independent component analysis, to GMV variations in the frontal and cerebellum regions and candidate risk single nucleotide polymorphisms (SNPs) data from 341 unrelated adult participants, including 167 individuals with ADHD, 47 unaffected siblings, and 127 healthy controls. We identified one SNP component significantly associated with one GMV component in superior/middle frontal regions and replicated this association in 317 adolescents from ADHD families. The association was stronger in individuals with ADHD than in controls, and stronger in adults and older adolescents than in younger ones. The SNP component highlights 93 SNPs in long non-coding RNAs mainly in chromosome 5 and 21 protein-coding genes that are significantly enriched in human neuron cells. Eighteen identified SNPs have regulation effects on gene expression, transcript expression, isoform percentage, or methylation level in frontal regions. Identified genes highlight MEF2C, CADM2, and CADPS2, which are relevant for modulating neuronal substrates underlying high-level cognition in ADHD, and their causality effects on ADHD persistence await further investigations. Overall, through a multivariate analysis, we have revealed a genomic pattern underpinning the frontal gray matter variation related to working memory deficit in ADHD.

## Introduction

Attention-deficit/hyperactivity disorder (ADHD) is a neurodevelopmental disorder characterized by pervasive symptoms of inattention and/or hyperactivity/impulsivity that affect normal functioning and development [1]. About 9.8% of children aged 3–17 years are estimated to have ADHD in the United States [2]. Based on long-term follow-up studies, 78% of boys [3] and 77.1% of girls [4] with ADHD showed persistence of ADHD into adulthood. Beyond symptoms, cognitive impairments are common in adults with ADHD, as indicated by studies of IQ [5], working memory [6, 7], response inhibition [8], executive functions [9], and educational outcomes [3, 4, 10]. The persistence of ADHD symptoms and associated cognitive impairments significantly elevates the risk of adverse outcomes for individuals with ADHD and increases the burden for the involved families and the whole society. Neuronal and genetic determinants of ADHD in adults remain largely unknown. Characterizing the underlying biological mechanisms would help delineate the pathology of ADHD and aid early interventions.

Converging evidence from anatomical studies suggest that children/adolescents with ADHD have global gray matter volume (GMV) reductions [11–15] and regional GMV reductions in the basal ganglia [12, 16–18], the frontal lobe [13, 18–20], the anterior cingulate cortex [21], and the cerebellum [11, 14, 18, 22]. Adults with ADHD also showed reduced total (cortical) GMV [23–25], reduced GMV in the frontal lobe [23, 25–28], caudate nucleus [29, 30], and cingulate cortices [16, 25, 27, 31], but the reduction is less conclusive as the recent ENIGMA-ADHD studies revealed no significant ADHD versus control differences for adults [32, 33]. Meanwhile, studies on ADHD-related persistent brain alterations are scarce, and the reported findings are inconsistent[30, 34].

Despite the persistent symptom and cognitive impairments in ADHD patients, the associated brain substrates remain understudied and inconsistent. Smaller GMV in the cerebellum was related to severe ADHD symptom (especially inattention) in children, adolescents [35, 36] and adults (18–63 years old) [28, 36] with ADHD. While in elderly (≥65 years old) patients with ADHD, greater GMV in the left cerebellum was associated with severer symptom [23]. Although the fronto-parietal network is well documented underlying working memory functions [37–45], its involvement in ADHD-related working memory deficit is understudied and inconclusive. Smaller GMV in the frontal region was related to poorer working memory performance in adults with ADHD [28]. But this relationship was absent in children with ADHD. Instead, smaller GMV [46] and thinner cortical thickness [47] in the temporal region were associated with poorer working memory performance in children with ADHD. The inconsistent results may be due to small sample sizes, methodological differences, and heterogeneity of the disorder [48, 49]. Recently, we leveraged structural magnetic resonance imaging (sMRI) data from reasonable large adult and adolescent samples to study brain alterations underlying persistent ADHD symptoms and cognitive impairments using consistent analytical approaches. We identified that GMV of the superior/middle/inferior frontal regions was consistently associated with working memory performance, and cerebellum GMV was consistently related to inattention symptoms in both adults and adolescents with ADHD [22, 28, 36]. Our findings emphasize that not only the symptom and impaired cognitive functions but also the underlying neurological alterations persist from childhood to adulthood in individuals with ADHD.

Since ADHD is highly heritable (estimated heritability: 77–88% [50]) and 12 independent single nucleotide polymorphism (SNP) loci showed significant differences between individuals with ADHD and controls in a recent ADHD genome-wide association study (GWAS) [51], ADHD susceptibility at the genomic level might underlie persistent brain alterations. A comprehensive ADHD imaging-genetic review paper summarized that thirteen candidate genes might underlie brain alterations of ADHD, while most existing studies only focused on a single gene or a few candidate genes in a specific pathway (e.g., dopamine-related pathway) [52]. Candidate gene-based analysis works fine if the prior knowledge is solid and well supported, but it cannot provide an overall picture of genes collectively affecting brain structure/function in ADHD. In contrast, polygenic risk score (PRS)-based analyses allow evaluations of the relationship between the overall genetic risk of disease and altered brain patterns. Recently, we examined the associations between PRS of ADHD and brain alterations in frontal and cerebellum regions that related to working memory performance and inattention in adults with ADHD [53]. We did not find any linear relationships between PRS and these brain alterations, although the ADHD-PRS was associated with hyperactivity symptoms, indicating that the lumpsum risk from the whole genome may not have the specificity to link to ADHD-related brain alterations.

**Fig. 1:**
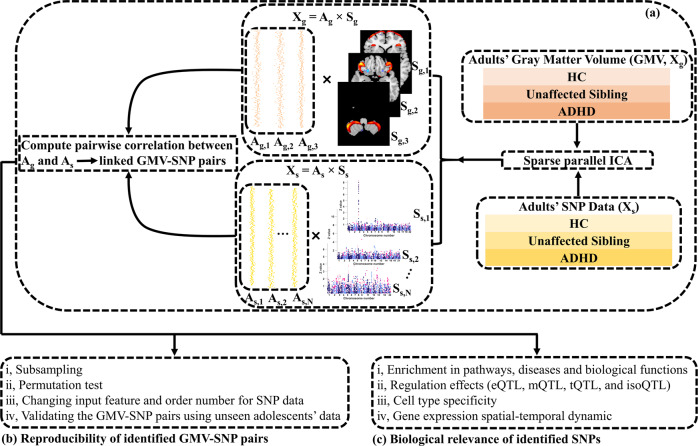
Analysis pipeline of the study. **a** Applying spICA to GMV and SNP data of 341 unrelated adult participants to identify linked GMV-SNP pairs. **b** Examining the stability and replicability of the identified GMV-SNP pairs by various robustness analyses and external validation. **c** Performing a series of downstream analyses to investigate the biological relevance of the identified SNPs.

Multivariate data-driven imaging-genetic fusion approaches may pose a promise for delineating the genetic factors underpinning GMV alterations. These approaches study a comprehensive set of SNPs and brain voxels, aiming to strike a balance between a few candidate variables and all-in-all lumpsum scores. They extract factors in a data-driven manner and assess associations at the multivariate factor level, which reduces the number of tests and boosts statistical power. Sparse parallel independent component analysis (spICA) [54], as one of the sparsity-regularized multivariate data fusion approaches, shows great promise for imaging genetics: spICA identified stable and replicable imaging-genetic pairs from the whole-brain sMRI and whole-genome SNP data of 35,692 adult participants in the UK biobank [54]. Here, spICA was performed on GMV and SNP array data of 341 unrelated adults aggregated from the NeuroIMAGE [55] and IMpACT-NL [7, 56] projects (Fig. 1a) and replicated on data of 461 adolescents from the NeuroIMAGE project [55]. Specifically, we investigated three gray mater networks in superior/middle/inferior frontal and cerebellum regions [28, 36], previously reported to be associated with working memory impairments (superior/middle/inferior frontal region) and inattention (cerebellum) in both adults and adolescents with ADHD, together with a set of SNPs that moderately discriminate individuals with ADHD from controls as implicated in the recent ADHD GWAS study [51].

Using ADHD adult samples for discovery exploration enables us to probe the genetic underpinning of gray matter variations in the frontal and cerebellar regions that are associated with persist ADHD symptom and cognitive impairment. Replicating the results in ADHD adolescent samples will potentially facilitate the identification of the timeline when the genetic factors exert the effects on altering gray matter, which is relevant to ADHD persistence.

## Materials and methods

### Discovery dataset

The discovery cohort was composed of 341 non-related European Caucasian adults (age: 18–63 years) aggregated from two projects: 198 samples from the NeuroIMAGE project [55] and 143 participants from the IMpACT-NL project [7, 56], including 127 healthy controls (HCs), 167 participants with ADHD and 47 unaffected siblings (unaffected siblings and ADHD cases were from different families). The IMpACT-NL project was approved by the regional ethics committee (Centrale Commissie Mensgebonden Onderzoek: CMO Regio Arnhem–Nijmegen; Protocol number III.04.0403). The NeuroIMAGE study was approved by the same regional ethics committee (2008/163; ABR: NL23894.091.08) and the medical ethical committee of the VU University Medical Center. Written informed consent was collected from all included participants.

All participants had an IQ >70, and did not have a psychotic disorder, addictions in the past 6 months, a current major depression, a diagnosis of autism spectrum disorder, epilepsy, neurological disorders, sensorimotor disabilities, or any medical or genetic disorders which might be confounded with ADHD [55–57]. The inclusion criteria for adult ADHD were slightly adapted (see details later) [55, 58] from the DSM-IV (NeuroIMAGE project) or DSM-IV-TR (IMpACT-NL). In addition, the presence of ADHD in childhood was required. Specifically, the 18 DSM-IV symptoms were assessed in all participants to evaluate their inattention and hyperactivity-impulsivity. Each symptom outcome ranged from 0 to 9, and the larger the value, the more severe the disorder. In a nutshell, adult participants with ADHD had five or more scores in the inattention and/or hyperactivity-impulsivity domain [55, 58]. Unaffected siblings were selected to have a score less than five in both the inattention and hyperactivity-impulsivity domains. HCs were screened to have a score less than 2 on total symptom counts [28]. See more details on the discovery sample enrollment and working memory performance in supplement S1. Table 1 lists the demographics and characteristics of 341 adult participants.

**Table 1:** Demographics and characteristics of the adult sample.

Variable	Diagnosis group (#)		
	ADHD (167)	Unaffected siblings (47)	Controls (127)
Age in years^a^	25.44 ± 8.65	20.91 ± 2.08	29.68 ± 12.35
Sex (male)^b^	95 (57%)	24 (51%)	36 (28%)
Estimated IQ^a^	103.81 ± 16.48	103.60 ± 12.71	108.61 ± 15.36
Inattention^a^	7.19 ± 1.74	1.72 ± 2.10	0.52 ± 1.18
Hyperactivity-impulsivity^a^	5.86 ± 2.37	1.51 ± 1.59	0.70 ± 1.00
Forward digit span score^a^	8.89 ± 1.98	9.00 ± 1.74	9.59 ± 1.93
Backward digit span score^a^	6.42 ± 2.31	5.96 ± 2.10	7.45 ± 2.04
History of stimulants^b^	78 (47%)	0	1 (0.8%)
Scan site (Nijmegen)^b^	115 (69%)	21 (45%)	108 (85%)
Scan site (Amsterdam)^b^	52 (31%)	26 (55%)	19 (15%)

Note, ^a^Data represented as mean ± standard deviation.

^b^Data is represented as a number (percentage).

### Replication dataset

The replication dataset included 461 European Caucasian adolescents (144 HCs, 129 unaffected siblings, and 188 ADHD) from 309 families (age: 7–17 years) recruited in the NeuroIMAGE project [55]. All participants provided written informed consent. Out of the 461 subjects, 452 were over 10 years old, and 403 were over 12 years old. Thus, we named this group adolescents throughout the whole paper. The exclusion criteria, evaluations of inattention and hyperactivity/impulsivity symptoms, assessments of working memory performance, as well as the grouping criteria for controls were the same as for adults in the aforementioned discovery cohort (see more details in supplement S2). The only difference was that adolescent participants with ADHD were required to have a score ≥6 in inattention and/or hyperactivity/impulsivity, and unaffected siblings had scores <6 in both symptom domains. Supplemental Table S1 lists the demographics and subject characteristics of 461 adolescents.

### sMRI data preprocessing

T1-weighted MRI images of both the discovery and replication samples were collected with 1.5T scanners with comparable settings across projects. Extensive quality controls have been performed on all T1 images using the method described in [28, 55, 56] and metrics derived from MRIQC. The included images were segmented into six types of tissues using SPM 12 (https://www.fil.ion.ucl.ac.uk/spm/software/spm12/) with the default tissue probability map (for adults) or a customized tissue probability map (for adolescents) generated by TOM 8 [36, 59]. Subsequently, gray matter images were normalized into Montreal Neurological Institute space, followed by modulation and smoothing with a 6 × 6 × 6 mm^3^ Gaussian kernel [36]. Further quality control, gray matter refinement (see below), and voxel-wise confounding effect (i.e., age, sex, and site, if applicable) adjustment were performed separately on adults and adolescents. After quality control, only those subjects with a correlation >0.8 with the mean gray matter map were kept. Gray matter refinement (i.e., masking) selected voxels with a mean gray matter volume >0.2 for further analyses, yielding 456,921 voxels for adults and 479,770 voxels for adolescents. 441,258 common voxels between adults and adolescents were used.

Identifying the genomic factors underlying GMV variations in the superior/middle/inferior frontal and cerebellum was our ultimate goal. Thus, we reconstructed GMV data of adults and adolescents to only include variations from these three regions of interest (ROIs, Fig. S1, ICs 2–4) [28, 36]. See more details about scanners, quality control, and the reconstruction of GMV data in supplement S3.

### Genetic data preprocessing

Both the NeuroIMAGE and the IMpACT-NL project used the Illumina Psych Array to genotype DNA extracted from blood. Quality control and imputation were then performed. All samples included in this study were European Caucasians. We further controlled subgroup differences by using five genomic ancestry components in later analyses. See supplement S4 for details on genetic data preprocessing.

Guided by a recent large GWAS study on ADHD [51] and the simulation results of spICA [54, 60], we selected 2108 SNPs by focusing on SNPs showing promising ADHD vs. HC difference (*p* < 1 × 10^−3^) in the GWAS summary statistics [51], and applying a light linkage disequilibrium (LD) pruning (*r*^2^ < 0.9 for p-value informed clumping). Light pruning does not inflate our statistical test, as correlated SNPs end up together in the same component after ICA factorization/decomposition [61].

### Identification of linked GMV-SNP pairs and examination of their replicability

We first applied spICA to the reconstructed GMV and risk SNP data from 341 unrelated adults to identify linked GMV-SNP pairs and then comprehensively examined their stability and replicability.

#### Identification of linked GMV-SNP pairs in discovery dataset using spICA

We performed spICA to decompose 341 adults’ reconstructed GMV and SNP data into three independent GMV components and 37 independent SNP components (Fig. 1a). Multivariate spICA decomposes GMV (**X**_**g**_) and SNP (**X**_**s**_) data into the product of a loading matrix (**A**_**g**_, **A**_**S**_ for GMV and SNP, respectively) and a component matrix (**S**_**g**_,**S**_**s**_ for GMV and SNP, respectively, **X**_**g**_ = **A**_**g**_×**S**_**g**_, **X**_**s**_ = **A**_**s**_ × **S**_**s**_). Each row of the component matrix (**S**_**g**_ or **S**_**s**_) is one independent brain/genomic component and each brain/genomic component is statistically independent of other brain/genomic components, thus enabling component/network-based analyses. Values in one brain/genomic component reflect the contributions of individual variables (voxels/SNPs) to the brain/genomic component. Each column of the loading matrix (**A**_**g**_ or **A**_**s**_) is the loading vector, and values of the loading vector represent weights of the corresponding brain/genomic component across participants. The sparsity control in spICA regulates the number of major contributing variables for each brain and genomic component. The spICA code will be released in the Fusion ICA Toolbox (FIT, https://trendscenter.org/software/fit).

The GMV component number was set to three due to an expectation that the decomposed GMV components would resemble the three predefined ROIs (ICs 2–4 in Fig. S1). The sparsity regularizer was initialized as one. No sparsity constraint was imposed on GMV data since signals can be easily separated from backgrounds for these three ROI priors. The Hoyer constraint threshold of SNP data was set as 0.4 based on the estimation strategy described in our previous paper [54]. The SNP component number was estimated based on Chen’s consistency measure [62]. Ten runs of spICA were performed, and ICASSO [63] was employed to select the most stable run to calculate GMV-SNP associations. Bonferroni correction was applied at *p* < 0.05 for comparison of 3*37 GMV-SNP associations.

#### Replicability of identified GMV-SNP pairs

Replicability of identified GMV-SNP pairs (Fig. 1b) was examined by (i) subsampling and permutation tests, (ii) changing input feature (applying different pruning and preselection p-value thresholds) and order (i.e., component) number for SNP data, (iii) validating identified GMV-SNP pairs using a replication dataset consisting of sMRI and SNP data of 461 adolescents recruited by the NeuroIMAGE project [55], and (iv) performing univariate genetic association analysis of the identified GMV component.

##### Subsampling and permutation tests

To test the stability of the identified GMV-SNP pairs, spICA was performed on 100 subsampled sets (Fig. 1b), where stratified sampling was employed to randomly select 90% subsamples from each of the ADHD, HC, and unaffected sibling groups. To test the likelihood of overfitting for the identified GMV-SNP associations, 1000 random permutations of the subjects were performed separately in the GMV and SNP data (Fig. 1b). We then applied spICA to the permuted data, and a null distribution was obtained based on the resulting pairwise associations of loadings. Then a p-value was computed as the percentage of pairs yielding significant GMV-SNP associations (Bonferroni correction was applied at *p* < 0.05 for 1000*37*3 pairs), which reflected the probability of overfitting of the spICA model.

##### Varying input features and order numbers for SNP data

We further checked the stability of the identified SNP component under two scenarios (Fig. 1b): (1) the number of SNP component (i.e. order number of SNP data) varied from 5 to 60 (SNP data were fixed with *p* < 1 × 10^−3^, *r*^2^ < 0.9 preselection); (2) SNP preselection *p*-value varied from 10^−4^ to 10^−2^ (*r*^2^ < 0.9) and the number of SNP component was estimated for each resulting SNP data according to Chen’s consistency measure [62]. The stability of the identified GMV-SNP associations (Fig. 1b) was also examined by performing spICA on heavily pruned SNP data (*r*^2^ = 0.2, *p* < 1 × 10^−3^ preselection).

##### Validation of linked GMV-SNP pairs in the replication dataset

To investigate the identified discovery GMV-SNP pairs in the replication dataset (Fig. 1b), we projected the identified GMV and SNP components into adolescent GMV and SNP data to obtain the corresponding loadings [64] (see supplement S5 for details on the projection method), and examined their associations using a mixed-effect linear regression model (model 5 in section 2.6). A false discovery rate (FDR) at *p* < 0.05 was applied for multiple comparisons of identified GMV-SNP pairs.

##### Validation of linked GMV-SNP pairs in different age groups

To investigate whether participants from different age groups presented similar GMV-SNP associations within the 317 adolescents from ADHD families (i.e., 188 cases and 129 unaffected siblings) in the replication dataset, we evaluated GMV-SNP associations in five subgroups partitioned by age distribution (as listed in Table S2), separately.

##### Univariate genetic association of the identified GMV component

We performed univariate association analyses between individual SNP loci and loadings of the identified GMV components, controlling for five genomic ancestry components, and reported the univariate association p-values in the Manhattan plot, which was compared to the identified SNP component via visual inspection.

### Statistical association analyses

For the discovery data (unrelated adults’ data), the associations among GMV components, SNP components, working memory, and symptom scores were examined using the following linear regression models:

1. a GMV component loading = a SNP component loading + five genomic ancestry components.
2. working memory or symptom variable = age + sex + a GMV component loading.
3. working memory or symptom variable = age + sex + a SNP component loading + five genomic ancestry components.
  In model 1, each GMV/SNP component was tested separately. Age and sex effects were not considered for GMV components in model 1 because they have been regressed out voxel-wisely in the preprocessing step (the same for models 5 and 6 below). Working memory performance was measured by maximum forward and backward digit span count. The symptom variable included inattention and hyperactivity/impulsivity symptoms. The ADHD cases vs. HCs difference of each GMV component was evaluated using a two-sample t-test, and the ADHD cases vs. HCs difference of each SNP component was assessed using the regression model 4:
4. a SNP component loading = diagnosis (ADHD case/HC) + five genomic ancestry components.
  For the replication data, family structure and medication history (binary values) were considered in the association analyses, as medication did affect the three brain ROIs studied here in adolescents included in this study [36]. Thus, the association between a GMV component and an SNP component and the ADHD cases vs. HCs difference of a GMV/SNP component were tested using the following linear mixed-effect models:
5. a GMV component loading = a SNP component loading + five genomic ancestry components + family ID + medication status.
6. a GMV component loading = diagnosis (ADHD case/HC) + medication status + family ID.
7. a SNP component loading = diagnosis (ADHD case/HC) + family ID + five genomic ancestry components.
  In models 5–7, the family structure was modeled as a random effect, and other predictors, i.e., an SNP component loading, diagnosis, medication, and five genomic ancestry components, were treated as fixed effects. Unaffected siblings were not used for ADHD cases vs. HCs comparison.
  Confounding effects, including medication, history of major depressive disorder (MDD), IQ, and diagnosis group (for GMV-SNP association), were examined for both discovery and replication data by adding them as a covariate, one at a time, to the models above. A previous study [56] reported that adult ADHD patients with previous MDD showed smaller hippocampus volume compared to adult ADHD patients without previous MDD. Part of the samples in [56] was included in this study (see details in supplemental material S1 and S2). So, we controlled the history of MDD to rule out any potential confounding effects from previous MDD.
  The model to test the interaction between the family label and SNP loading for predicting GMV IC 1 loading by controlling five genomic ancestry components is listed below:
8. GMV IC 1 loading = SNP loading + five genomic ancestry components + family label (ADHD/HC family) + family label*SNP loading

In model 8, the family label was coded as a binary variable.

### Interpretation of identified GMV and SNP components and their biological relevance

Each of the identified GMV and SNP components was normalized to have a zero mean and unit standard deviation. GMV component regions were selected with $|z| > 2.5$ and mapped into the Talairach atlas [65].

SNPs with absolute weights larger than two were selected for further examinations of their biological relevance (Fig. 1c), including (i) gene annotation and enrichment in pathways, diseases, and biological functions by using Ingenuity Pathway Analysis (IPA, QIAGEN Inc., https://www.qiagenbioinformatics.com/products/ingenuity-pathway-analysis) and Gene Ontology (http://geneontology.org/), (ii) regulation effects in prefrontal regions via expression quantitative trait loci (eQTL), transcript expression via transcript QTL (tQTL), isoform percentage via isoform QTL (isoQTL) based on the summary statistics available on the PsychENCODE website (http://resource.psychencode.org/) and methylation via methylation QTL (mQTL) based on Jaffe’s study [66], (iii) enrichment of annotated genes in five human brains cell-type-specific gene sets based on Zhang’s study [67], (iv) gene expression temporal dynamic in the dorsolateral prefrontal cortex based on BrainSpain RNA-seq data [68–70].

## Results

### Discovery results

**Fig. 2:**
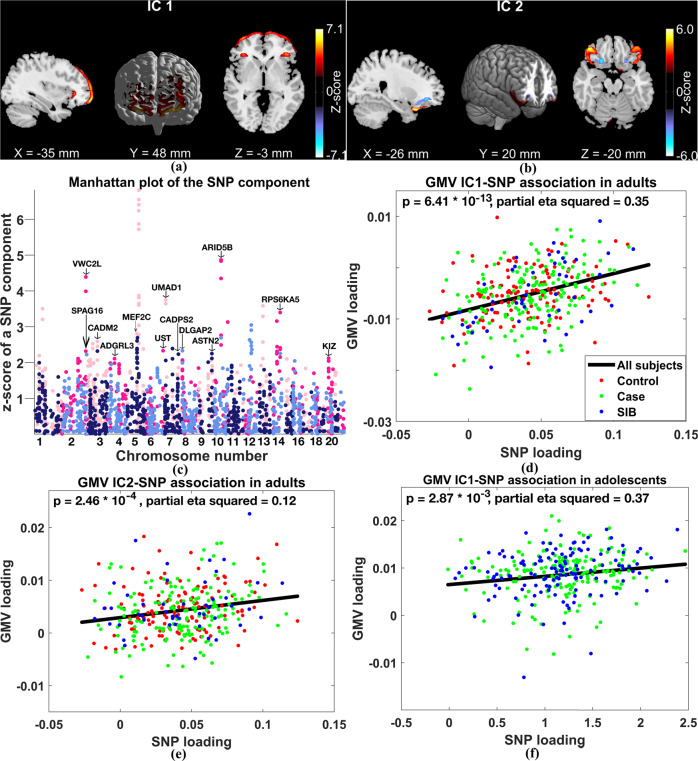
Results of spICA in discovery and replication sets. The identified (**a**) GMV IC 1, (**b**) GMV IC 2, and (**c**) SNP component. The association between GMV IC 1 and SNP component was (**d**) significant in adults (the discovery set) and (**f**) replicated in adolescents (the replication set). **e** The association between GMV IC 2 and SNP component was significant in adults. In subplots **a**–**c**, hot colors represent positive weights, and cold colors denote negative weights. In subplots **d**–**f**, black, red, green, and blue colors represent all subjects, controls, cases, and unaffected siblings (SIB).

For the discovery data, spICA identified that two GMV components (IC 1 and IC 2 in Fig. 2a, b) showed significant and positive associations with one SNP component in Fig. 2c (IC 1-SNP_*p* = 6.41 × 10^−13^, IC 1-SNP_$\eta _p^2$ (partial eta squared) = 0.35; IC 2-SNP*_p* = 2.46 × 10^−4^, IC 2-SNP_$\eta _p^2$ = 0.12) after controlling for population structure and applying Bonferroni correction (p-value threshold was 0.05/(3*37)). Among 100 stratified subsampling tests, 89 subsamples stably identified GMV IC 1-SNP and GMV IC 2-SNP pairs with similar correlation strengths as in the full sample. 1000 permutation tests generated tail probabilities of *p* = 4.10 × 10^−2^ for GMV IC 2-SNP and *p* = 1.20 × 10^−2^ for GMV IC 1-SNP. Subsampling and permutation results supported that the identified GMV-SNP associations were stable in adults, and the corresponding correlation coefficients were likely not overfitted. Positive associations between GMV ICs 1–2 and the identified SNP component indicated that more counts of minor/reference/effect alleles in SNPs with positive weights (hot-colored loci in Fig. 2c) were related to higher GMV in brain regions with positive weights (hot-colored regions in Fig. 2a, b), and vice-versa. Figure 2d, e plot the associations between loadings of the SNP component and loadings of GMV IC 1 and IC 2, respectively. We observed that the GMV IC 1-SNP component association was much stronger in unaffected siblings and ADHD patients compared to controls (sibling_$\eta _p^2$ = 0.60, ADHD_$\eta _p^2$ = 0.43, control_$\eta _p^2$= 0.17). GMV IC 3-SNP component association (supplemental Table S3) was not significant in discovery samples (*p* = 0.56).

Further, we treated subjects from ADHD families (i.e., cases and unaffected siblings) as one group and participants from control families as another group. We observed that this categorical family label significantly interacted with SNP loading for GMV IC 1 prediction (*p* = 8.50 × 10^−3^) using model 8 in section 2.6. Diagnosis group, medication, IQ, and comorbidity did not confound the identified GMV-SNP associations.

We confirmed that the identified SNP component was robust to a range of order numbers for SNP data (SNP component number range: [24, 40]) and SNP preselection p-values (*p*: [0.0005, 0.005]). Applying spICA to the heavily pruned SNP data (*r*^2^ = 0.2, *p* < 1 × 10^−3^), we identified a similar pair as reported in the main finding (the GMV IC 1-SNP pair), indicating that the discovered GMV IC 1-SNP association was unlikely biased by LD structure. See supplement S6 for details.

### Replication results

In 461 adolescents, GMV IC 1-SNP component association was nominally significant after controlling for medication and five genomic ancestry components (uncorrected *p* = 4.20 × 10^−2^, $\eta _p^2$ = 0.17). GMV IC 2-SNP component association was not replicated. Similarly, we observed a stronger association for GMV IC 1-SNP component in unaffected siblings compared to ADHD patients and controls; the GMV IC1-SNP component associations in three groups were sibling_$\eta _p^2$ = 0.73, ADHD_$\eta _p^2$ = 0.29, control_$\eta _p^2$= 0.31. Moreover, the categorical family label (ADHD/HC) significantly interacted with the SNP loading for GMV IC 1 prediction (*p* = 4.83 × 10^−3^) with controlling for medication effect. Focusing on adolescents from ADHD families, the association between GMV IC 1 and SNP component became significant (Fig. 2f, corrected *p* = 5.74 × 10^−3^, $\eta _p^2$ = 0.37, corrected for two GMV-SNP pairs identified in the discovery samples). The association between GMV IC 2 and the SNP component remained not significant. Moreover, GMV IC 1-SNP component association was significant in 165 older (15–17 years) adolescents (corrected *p* = 4.60 × 10^−2^, $\eta _p^2$ = 0.42) but not in 152 younger ones (age: 7–15 years, *p* = 0.20). See more results of sub-age adolescent groups in supplement S7. The diagnosis group, IQ, and major depression did not confound the association between GMV IC 1 and the SNP component in adolescents from ADHD families. GMV IC 3-SNP component association (supplemental Table S3) was not significant in 461 adolescents (*p* = 0.80), also not significant in 317 adolescents from ADHD families (*p* = 0.17).

### Genetic association analysis of the GMV loading

None of the 2108 SNPs showed a significant association with GMV IC 1 after FDR correction. However, the p-value map of the association highlighted SNP loci in chromosomes 5, 1, and 9, which were largely consistent with the identified top SNPs. See supplement S8 for details.

### Brain region identification

The identified GMV IC 1 (z-scored) is illustrated in Fig. 2a, highlighting superior and middle frontal gyri (|z| > 2.5). GMV IC 1 did not show significant ADHD versus control difference in adults but showed significant GMV reduction in adolescents with ADHD after controlling for the medication effect (*p* = 8.72 × 10^−3^, *t* = 2.64, DF = 329). Loadings of GMV IC 1 were significantly and positively associated with backward (*p* = 2.09 × 10^−2^,$\eta _p^2$ = 0.03) and forward (*p* = 2.41 × 10^−2^,$\eta _p^2$ = 0.10) digit span performance in adults and adolescents, respectively.

### Highlighted SNPs identification, their annotations, and biological relevance

Figure 2c shows the absolute values of the z-scored SNP component. The identified SNP component showed no significant ADHD cases vs. HCs difference and was not significantly associated with forward/backward digit span performances or symptom scores in both discovery and replication sets.

The identified SNP component highlighted 93 top SNPs (|z| > 2, see supplemental spreadsheet). Using the human hg 19 build, 93 top SNPs were mapped to long non-coding RNAs (lncRNAs) mainly in chromosome 5, and 21 protein-coding genes, which were not significantly enriched in any particular pathways of Gene Ontology. Performing IPA on the 21 protein-coding genes, five genes, including MEF2C, DLGAP2, CADPS2, CADM2, and ADGRL3, were identified as being involved in cell-to-cell signaling and interaction (*p*-value range: [2.24 × 10^−4^, 4.18 × 10^−2^]). Six genes, i.e., MEF2C, DLGAP2, CADPS2, CADM2, ADGRL3, and UST, played a role in the nervous system development function (*p*-value range: [2.24 × 10^−4^, 4.18 × 10^−2^]). In addition, MEF2C, DLGAP2, and CADPS2 exerted effects on excitatory postsynaptic potential (*p* = 2.24 × 10^−4^).

Out of the 93 top SNPs, 4 acted as cis-eQTLs of three protein-coding genes and one lncRNA (Table S4), 6 were cis-isoQTLs of five unique transcripts (Table S5), and 3 were cis-tQTLs of three unique transcripts (Table S6). Moreover, 10 SNPs significantly regulated methylation levels of 9 unique CPG sites (Table S7). See supplement S9 for details of regulation effects. Furthermore, the annotated 21 genes were significantly enriched in human brain neuron cells (*p* = 0.02). Take MEF2C and CADPS2 as examples; their expression level in DLPFC from early fetal to middle adulthood is presented in supplement S10.

## Discussion

In this study, we applied spICA to GMV and SNP data from an ADHD adult cohort, and we identified one SNP component significantly and positively associated with two GMV components (GMV ICs 1–2 in Fig. 2a, b). The association between SNP and GMV IC 1 was further replicated in adolescents from ADHD families, but with overall weaker strength compared to adults. Meanwhile, within adolescent samples, the GMV IC 1-SNP association was stronger in older participants than in younger ones, indicating that the identified genetic component consistently exerted effects on perturbating GMV in the superior and middle frontal regions across development, but may exert their effects fully later in the development. Overall, participants from ADHD families carrying a higher load of this SNP component had larger gray matter volume in the superior and middle frontal gyri, and this relationship strengthened with age. We did not observe significant associations among control participants. Since individuals with ADHD showed no significant difference in GMV of superior and middle frontal gyri compared to controls in adults but demonstrated a significant reduction in adolescents, we speculate that a higher load of the identified SNP component likely compensates for GMV reduction in the frontal cortex in individuals with ADHD, and this compensation effect may be exerted fully in adulthood.

The identified superior/middle frontal regions are well documented in ADHD neuroimaging studies. Hoogman, et al. [33] reported that widespread cortical surfaces, including superior frontal, presented significantly reduced area in children with ADHD, and the reduction was attenuated in adolescents and adults in a large-scale ENIGMA-ADHD study. Zhao and colleagues [71] characterized GMV reduction in the left superior frontal and right middle frontal gyri in ADHD adolescents. Our results are in line with these findings showing that GMV reduction in superior and middle frontal gyri occurs in children/adolescents with ADHD but diminishes in adults with ADHD, suggesting a delayed maturation in the frontal cortex [72–75]. Better working memory performance has been related to higher GMV in the prefrontal region [76, 77] and larger surface area in superior and medial-orbital frontal gyri [45], which lends support to our results that higher GMV in superior and middle frontal gyri relates to better working memory performance in both adults and adolescents.

The top SNPs identified via spICA highlighted SNPs in lncRNAs in chromosome 5 and SNPs in 21 protein-coding genes, including MEF2C, CADPS2, and CADM2. The lncRNA transcript, RP11_6N13.1, has been associated with educational attainment [78] and broad depression [79], even though its biological function is unclear. MEF2C, highly expressed in DLPFC from early fetal to middle adulthood based on BrainSpan RNA-seq data [68–70], has a clear role in neuronal development and function [80–82]. For instance, mice were hyperactive and showed impaired motor coordination [83] after conditionally knocking out Mef2c. MEF2C mutations have been associated with ADHD [84], intellectual disability [85, 86], and other mental disorders[87–91]. Other important genes are CADM2 and CADPS2. They are both highly expressed in the frontal cortex [68–70, 92] and were associated with ADHD [93] and other mental disorders [94–99].

The findings presented here should be considered in context with their strengths and limitations. This study leveraged the recent ADHD GWAS study [51] to provide us with good candidates to investigate SNPs underlying brain alterations related to ADHD. However, samples from the NeuroIMAGE project included in this study were also utilized in the ADHD GWAS [51]. It is worth noting that the ADHD GWAS summaries were only used to select candidate SNPs as input for spICA and were not involved in the spICA analysis to identify SNP components related to brain patterns. This mitigates inflation due to overlapping samples. Another limitation is that the sample size of this study is relatively small compared to other genomic studies (e.g., GWAS), future work with a larger discovery and replication sample size is needed, particularly for replicating GMV IC2-SNP association. Another potential limitation is that our ADHD and control groups were not matched on age and sex. Thus, there might be residual confounding effects on case vs. control differences, even after we regressed them beforehand.

This study showed the first application of our novel spICA to a clinical population. This study, together with another study in the general population (i.e., UK Biobank) [54], indicated that spICA has great potential to reveal stable and replicable genomic features underlying brain alterations related to diseases or brain development. In the future, we expect to apply spICA to other clinical populations (e.g., schizophrenia, bipolar disorder, etc.) to uncover new genomic features relevant to the neurobiology of these diseases.

In summary, spICA identified that one SNP component related to GMV in superior and middle frontal gyri underlying working memory performance in adults and adolescents with ADHD. This association was more significant in ADHD families and in older participants than in controls and younger participants. The top contributing SNPs resided in lncRNAs in chromosome 5 and a set of genes that were enriched in human brain neuron cells. Altogether, we present a new application of our novel multivariate method, which allows new ways to link genome-neuroanatomical variation to uncover new features relevant to ADHD neurobiology.

## Supplementary information

Supplemental material

Annotation of the identified 93 top SNPs
