# Supplementary material for: Genomic patterns linked to gray matter alterations underlying working memory deficits in adults and adolescents with attention-deficit/hyperactivity disorder
Source: Transl Psychiatry. 2023 Feb 11;13:50. doi: 10.1038/s41398-023-02349-x (PMC9922257; doi:10.1038/s41398-023-02349-x)
Supplement: Supplementary file 1 — Supplemental material [file 41398_2023_2349_MOESM1_ESM.docx]

**Supplemental materials of the manuscript ‘Genomic patterns linked to gray matter alterations underlying working memory deficits in adults and adolescents with attention-deficit/hyperactivity disorder’**

**S1, Additional information on discovery sample**

NeuroIMAGE participants were enrolled at the Vrije Universiteit in Amsterdam or the Radboud University Medical Center in Nijmegen. IMpACT-NL participants were enrolled at the Radboud University Medical Center in Nijmegen. The presence of ADHD in childhood was retrospectively assessed in IMpACT-NL participants. NeuroIMAGE participants had a formal research diagnosis of ADHD in childhood. Out of 341 adults, 12 controls and 34 patients with ADHD had a history of major depressive disorder.

Working memory performance, including maximum digit span forward and backward counts from the WAIS Digit Span task [4], was consistently assessed in both projects. Significant ADHD vs. control differences have been observed for maximum digit span forward (*p* = 2.62×10^-3^, *t* = 3.02, degree of freedom (DF) = 287) and backward (*p* = 7.27×10^-4^, *t* = 3.96, DF = 287) counts in adults.

**S2, Additional information on replication sample**

188 out of 461 adolescents fulfilled the diagnosis of ADHD at the time of scanning. Out of 461 adolescents, 3 controls, 2 unaffected siblings and 2 patients with ADHD had a history of major depressive disorder. The 461 adolescent participants included 176 independent singletons, 116 pairs of siblings, 15 sets of three siblings, and 2 sets of four siblings. Significant ADHD vs. control differences were observed for maximum digit span forward (*p* = 1.77×10^-6^, *t* = 4.87, DF = 327) and backward (*p* = 1.90×10^-7^, *t* = 5.32, DF = 327) counts in adolescents.

**S3, Scanner information, MRIQC-based quality control, and the reconstruction of GMV data**

Two Siemens scanners with comparable settings were used in NeuroIMAGE and IMpACT-NL projects. NeuroIMAGE project utilized Siemens SONATA and Siemens AVANTO, and IMpACT-NL project employed Siemens SONATA.

For sMRI image quality check, we also computed the coefficient of joint variation (CJV) using MRIQC [5] of all images included. All images had CJV values within three standard deviations from the mean, except for two which had CJV values within four standard deviations away from the mean. Since we consider this amount of variability in CJV reasonable, no images were removed based on the CJV values.

Our previous work [6,7] highlighted that altered GMV in three networks, including regions of superior/middle/inferior frontal and cerebellum (ICs 2-4 in Figure S1), consistently related to working memory deficit or inattention in both adults and adolescents. We further confirmed that these regions showed no significant associations with head motion parameters (e.g., CJV, contrast-to-noise ratio, and entropy focus criterion) estimated from MRIQC [5], indicating a low likelihood of being driven by head motion. We reconstructed GMV data of adults and adolescents to only include variations from these three regions of interest (ROIs, Figure S1, ICs 2-4) that were identified using independent component analysis ($\mathbf{X}$ (GMV data) = $\mathbf{A}$ (loading matrix)$\boldsymbol{\times}$ $\mathbf{S}_{\mathbf{g}}$ (spatial map))) in the previous study [6,7]. Given the current whole-brain GMV data $\mathbf{X}_{\mathbf{g}}$ and the spatial maps $\mathbf{S}_{\mathbf{g}}$ of three ROIs, we computed the projected loading matrix $\mathbf{A}_{\mathbf{g}}$ of three ROIs on the current GMV data $\mathbf{X}_{\mathbf{g}}$ as $\mathbf{A}_{\mathbf{g}}=\mathbf{X}_{\mathbf{g}}\boldsymbol{\times}\mathbf{S}_{\mathbf{g}}^{\mathbf{+}}$**(**$\mathbf{S}_{\mathbf{g}}^{\mathbf{+}}$ denotes the pseudoinverse of $\mathbf{S}_{\mathbf{g}}$**)**, and then reconstructed GMV data ($\mathbf{X}_{\mathbf{gr}}$) as $\mathbf{X}_{\mathbf{gr}}=\mathbf{A}_{\mathbf{g}}\boldsymbol{\times}\mathbf{S}_{\mathbf{g}}$, which was then input to spICA.

**S4, Genetic data preprocessing:**

DNA extracted from blood was genotyped with the Illumina Psych Array for both NeuroIMAGE and IMpACT-NL projects. Pre-imputation quality control (QC) was performed to remove gender-mismatched samples and SNPs with minor allele frequency (MAF) < 0.01, call rate < 95%, and Hardy­ Weinberg Equilibrium < 1×10^-6^. Imputation was performed based on ENIGMA protocol with 1000 genome as the reference. Only SNPs with imputation r^2^ > 0.3 were included. We further removed participants with missing rate > 15% and SNPs with missing rate > 10% (yielding to 5,674,622 SNPs). Univariate case vs. control analysis was then performed; the obtained p values largely formed a uniform distribution (the corresponding Q-Q plot is shown in Figure S2). Samples included in this study fell into a homogenous group (i.e., European ancestry). And we controlled for subgroup differences by using five genomic ancestry components.

**S5, Projecting the identified GMV-SNP pairs to replication dataset**

Here we described the rationale of the projection method. Given that the discovery and replication datasets included participants of different age groups (the discovery dataset included adults and the replication dataset included adolescents), the strongest linked GMV-SNP patterns may be different for these two populations (no matter what imaging-genetic fusion methods were applied). That is because all these association-driven methods were designed to optimize the correlation between modalities. Thus, the pair identified in the discovery dataset might even be absent in the replication dataset. This could cause discovery components to be undetectable in the replication set using association-driven fusion approaches. Thus, to investigate the identified GMV-SNP pairs in the replication dataset, we used a projection method with the assumption that the replication dataset shared the same components.

The projection method can be described as: let $\mathbf{S}_{\mathbf{dg}}$ and $\mathbf{S}_{\mathbf{ds}}$ denote the source/component matrices of adults’ (discovery) GMV and SNP data, respectively. Let $\mathbf{X}_{\mathbf{rg}}$ and $\mathbf{X}_{\mathbf{rs}}$ represent adolescents’ (replication) GMV and SNP data, respectively. Then the corresponding loading matrix of the adolescents’ GMV data can be estimated as $\mathbf{A}_{\mathbf{rg}}=\mathbf{X}_{\mathbf{rg}}\boldsymbol{\times}\mathbf{S}_{\mathbf{dg}}^{\mathbf{+}}$. Recall that spICA utilized nonlinear sparsity regularization and reconstructed SNP data with cleaner sources at each iteration (i.e., denoising the SNP components of the discovery dataset). Thus, we reconstructed the loading matrix of SNP data using Tikhonov-regularized least squares [8] to account for the denoising effect in the replication SNP data. The loading matrix of SNP data was reconstructed as $\mathbf{A}_{\mathbf{rs}}\mathbf{=}\mathbf{(X}_{\mathbf{rs}}\text{+}\text{α}\text{I}\boldsymbol{)\times}\mathbf{S}_{\mathbf{ds}}^{\mathbf{+}}$ , where $\text{I}$ is an identity matrix, $\text{α}$ is a parameter calculated as in [9] to balance between retaining the signal and reducing the noise.

**S6, Results of replicability analyses**

***1, varying the number of SNP components from 5 to 60***

The identified SNP component in Figure 2(c) consistently showed up when the SNP component number varied from 19 to 60. The correlation coefficient between the identified SNP component and the matched one from different component order was between 0.57 and 0.70. The top 5 SNPs in the identified SNP component were stably positioned in the top 5 in all matched components. Figure S3 plots the overlap ratio of the top 5 SNPs when the SNP component number varied from 5 to 60. When the component number was between 5 and 18, the variance of the identified SNP component (Figure 2(c)) may be too small to be included due to dimension deduction, thus resulting in low detection accuracy.

***2, varying SNP preselection p-value from 0.0001 to 0.01***

According to Chen’s consistency measure, the SNP component numbers were estimated as 60, 37, 20, 50, 18 for SNP data with preselection p value threshold of 0.0001, 0.0005, 0.001, 0.005, 0.01, respectively. When SNP data preselection p-value varied from 0.0005 to 0.005, the identified SNP component consistently presented (the correlation between the identified SNP component and the matched one was between 0.53 and 0.67), and the top 5 SNPs in the identified SNP component largely held as top 5 in the matched SNP component (Figure S4). When *p* = 0.01, the correlation between the identified SNP component and the matched one is 0.44, and 3 out of 5 top SNPs were still top SNPs, which may be caused by the fact that too many SNPs were included when the preselection p-value was 0.01 and the small variance in the identified SNP component may be dropped after dimension reduction.

***3, Applying heavy pruning (r^2^=0.2) to SNP data***

When applying heavy pruning (r^2^=0.2) to the SNP data with preselection *p* < 1×10^-3^, one GMV component was significantly associated with one SNP component (*p* = 5.91×10^-12^ , $\eta_{p}^{2}$ = 0.19), where the GMV component was highly correlated with GMV IC 1 in Figure 2(a) (*r* = 1), and the SNP component had a correlation coefficient of 0.63 with the SNP component in Figure 2(c), and 4 out of 10 top SNPs were still presented as top SNPs in the matched one. The New 6 top SNPs may result from the fact that p-value informed clumping in Plink software retained SNPs showing more significant case vs. control difference instead of those with larger weights in the SNP component. Noteworthily, the identified GMV-SNP component pair from *p* < 1×10^-3^, *r*^2^ < 0.2 set was nominal significant in 317 subjects from ADHD families in adolescents (*p* = 3.76×10^-2^, $\eta_{p}^{2}$ = 0.09), indicating that the discovered GMV IC 1-SNP association was less likely biased by LD structure.

**S7, GMV-SNP associations in sub-age adolescent groups**

When the association was tested in sub-age groups, 165 older adolescents (age: 15-17 years) demonstrated significant positive GMV IC 1-SNP component association (corrected *p* = 4.60×10^-2^, $\eta_{p}^{2}$ = 0.42), and it was not significant in 152 younger ones (age: 7-15 years, *p* = 0.20). The GMV IC 2-SNP component association was not significant in both age groups (i.e., age range: 7-15 years and age range: 15-17 years). Similarly, GMV IC 1-SNP components association was nominally significant in the 118 oldest adolescents (age: 16-17 years, uncorrected *p* = 4.13×10^-2^, $\eta_{p}^{2}$ = 0.41, beta > 0) but not in younger ones (age: 7-14 years and age: 14-16 years). The GMV IC 2-SNP component association was not significant in the three age groups (age range: 7-14 years; 14-16 years, and 16-17 years).

**S8, Univariate genetic association analysis of the GMV loading**

Figure S5 displays the Manhattan plot of -log_10_(p) of the association between each SNP and loadings of GMV IC 1 in 341 adults, where interchange pink and dark pink colors were used to distinguish two sequential chromosomes. After FDR correction, none of the 2108 SNPs showed a significant association with GMV IC 1 (the same for the SNP dataset with heavy pruning of *r*^2^ = 0.2). However, the p-value map of the association highlighted SNP loci in chromosomes 5, 1, and 9, which were largely in line with the highlighted regions in the identified SNP component by spICA (Figure 2(c)) based on visual inspection.

**S9, Regulation effects of 93 top SNPs**

Based on summary statistics of eQTL, tQTL, and isoQTL for the prefrontal cortex available on the PsychENCODE website (http://resource.psychencode.org/), we identified that out of the 93 top SNPs, 4 acted as cis-eQTLs of three protein-coding genes and one lncRNA (Table S4), 6 were cis-isoQTLs of five unique transcripts (Table S5), and 3 were cis-tQTLs of three unique transcripts (Table S6). Importantly, SNP rs1313237 significantly and positively regulated the expression level of PCNXL4 gene, and negatively regulated the isoform percentage and expression level of transcript DHRS7-001 (protein-coding transcript). SNP rs2842198 significantly and positively regulated the expression level of TIE1 gene and the expression level of transcript TMEM125-001 (protein-coding transcript). SNPs rs373098 (positive regulation) and rs2362108 (negative regulation) significantly regulated the isoform percentage of transcript MEF2C-023. SNPs rs6047270 significantly and positively regulated the isoform percentage and expression level of transcript PLK1S1-007. Moreover, 10 out of 93 top SNPs significantly regulated methylation levels of 9 unique CPG sites (Table S7) using mQTL summary statistics in the human frontal cortex provided in [10]. In particular, SNP rs56144910 significantly and positively regulated the methylation level of cg18498987 located in the 5′ untranslated regions of MEF2C.

**S10, Expression of MEF2C and CADPS2 in DLPFC from early fetal to middle adulthood**

The expression level (log2 transformed) of MEF2C and CADPS2 in DLPFC from early fetal to middle adulthood is plotted in Figure S6. MEF2C was highly expressed in DLPFC throughout all age stages, especially before birth (with some fluctuations). After birth, its expression level was high before one year old and markedly dropped at two years old, followed by a steady increase between 2 and 11 years old, and finally reached a relatively stable (with some fluctuations) expression level between 12 and 40 years old. CADPS2 was mainly expressed in DLPFC after birth; its expression level gradually increased starting from 19 postconceptional weeks to 1 year old and dramatically dropped at two years old, followed by a steady increase from 2 to 11 years old, and arrived at a relatively stable expression level between 12 and 40 years old.


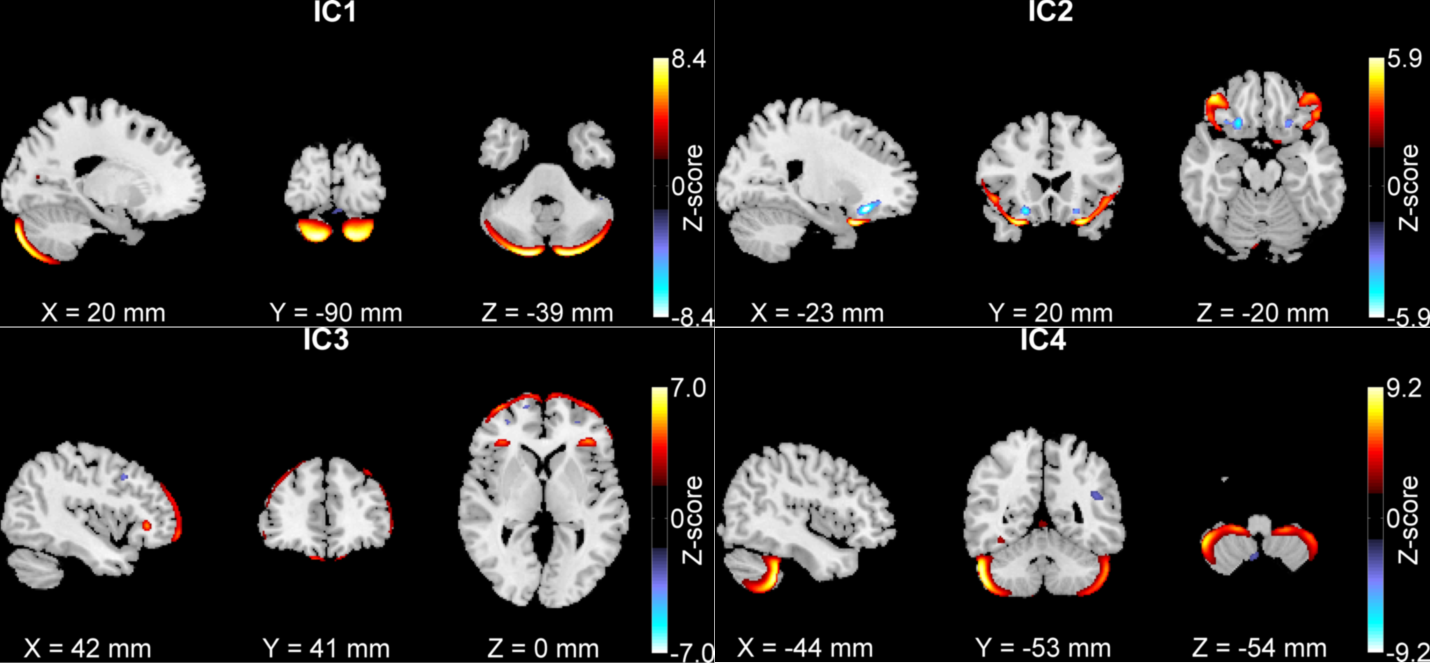


**Fig. S1.** Four GM components (ICs) are significantly associated with either working memory or inattention symptoms (|Z| > 2.5). ICs 2-4 are consistently associated with working memory performance/inattention in both ADHD adults and adolescents [11]. The figure comes from the paper [6].

**
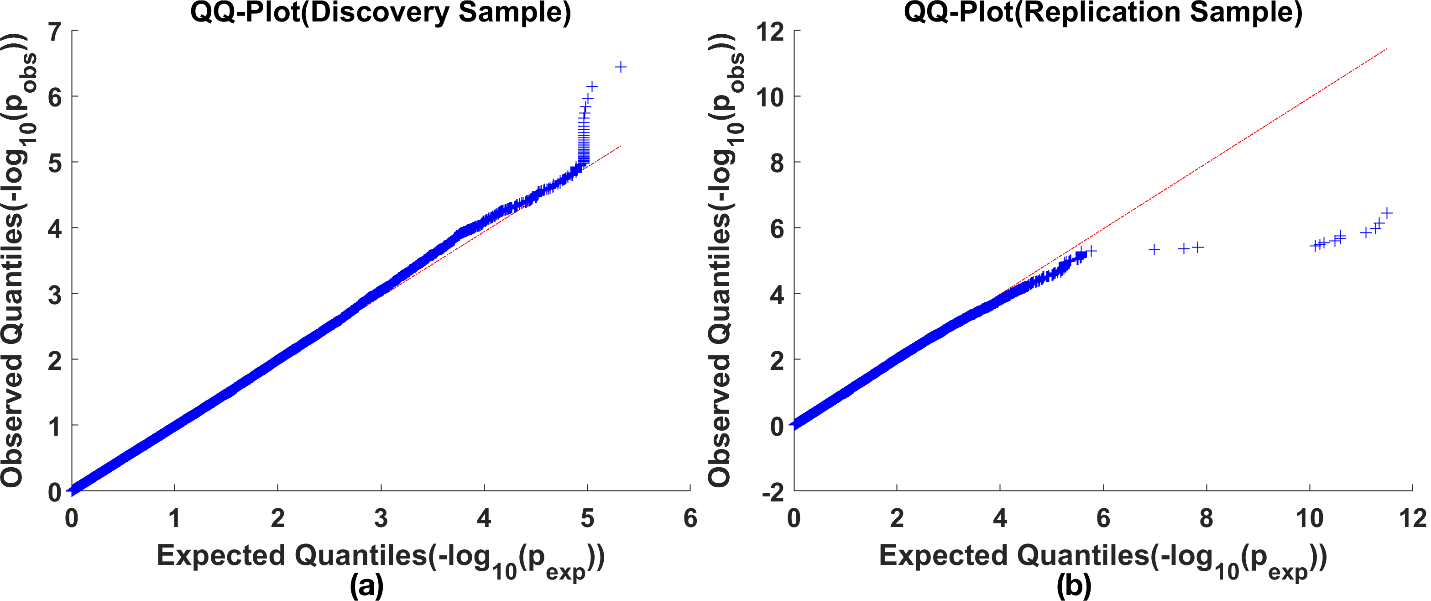
**

**Fig. S2**. Q-Q plots of sorted -log_10_(p_obs_) values (ascending order, p_obs_ values were obtained from univariate case vs. control MAF difference test) against sorted -log_10_(p_exp_) values (ascending order, p_exp_ were sampled from a uniform distribution) for (a) discovery and (b) replication samples.





**Fig. S3**. Top SNP overlap ratio when SNP component number varied from 5 to 60.





**Fig. S4**. Top SNP overlap ratio when SNP preselection p-value varied from 0.0001 to 0.01.

**
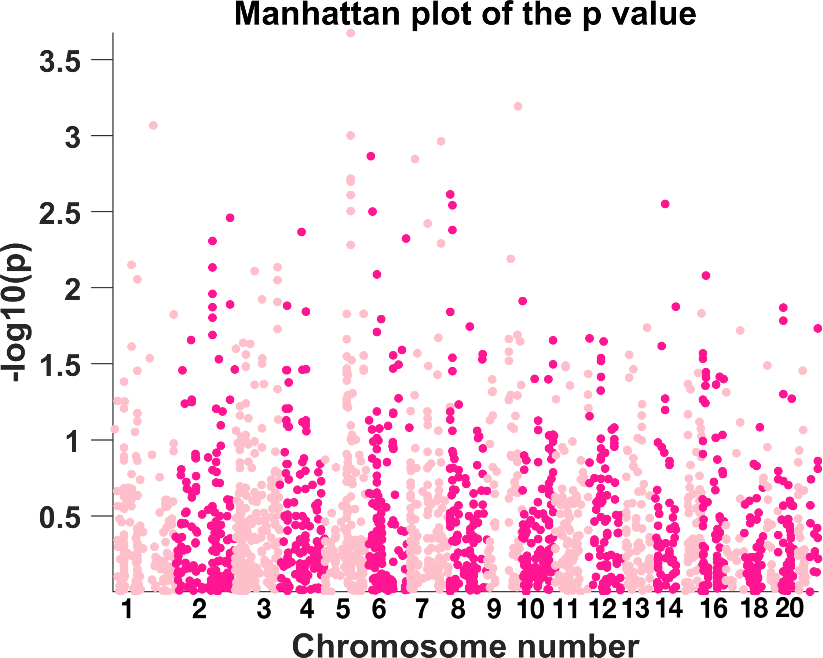
**

**Fig. S5**. Manhattan plot of -log_10_(p) of the association between individual SNP and loadings of GMV IC 1 in adults.


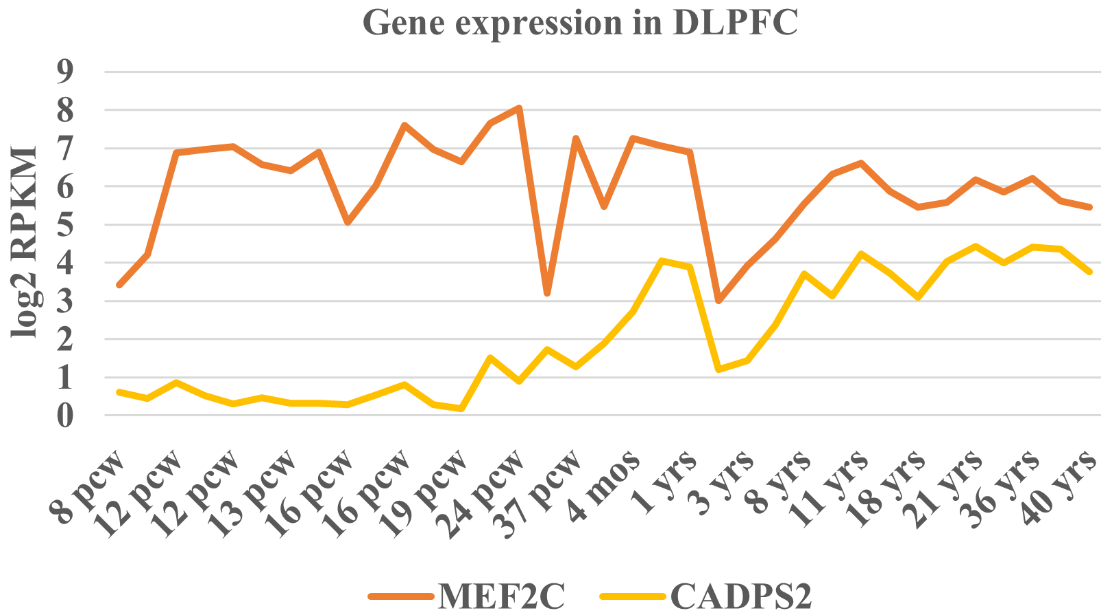


**Fig. S6**. The expression level of MEF2C (in red-orange color) and CADPS2 (in orange color) genes in the DLPFC region based on BrainSpan RNA-seq data [12-14]. The expression values (y-axis) were log2 transformed. RPKM denotes reads per kilobase million, and ‘pcw’, ‘mos’, and ‘yrs’ represent postconceptional week, month, and years old, respectively.

**TABLE S1.** Demographics and subject characteristics of adolescent participants

| Variable | Diagnosis group (#) | | | |  |
| --- | --- | --- | --- | --- | --- |
|  | ADHD (188) | Unaffected sibling (129) | Healthy control (144) | |  |
| Age in years ^a^ | 14.74 ± 2.32 | 14.92 ± 1.97 | 14.50 ± 2.17 |  | |
| Sex (male) ^b^ | 119 (63%) | 55 (43%) | 85 (59%) |  | |
| Estimated IQ ^a^ | 92.70 ± 16.09 | 99.22 ± 13.94 | 104.69 ± 13.54 |  | |
| Inattention ^a^ | 7.37 ± 1.69 | 1.35 ± 1.98 | 0.78 ± 1.71 |  | |
| Hyperactivity-impulsivity ^a^ | 5.87 ± 2.44 | 0.97 ± 1.55 | 0.38 ± 1.14 |  | |
| Forward digit span score ^a^ | 7.84 ± 1.76 | 8.63 ± 1.63 | 8.78 ± 1.70 |  | |
| Backward digit span score ^a^ | 4.95 ± 1.67 | 6.01 ± 1.72 | 6.07 ± 1.97 |  | |
| History of stimulants ^b^ | 106 (56%) | 11 (9%) | 1 (0.7%) |  | |
| Scan site (Amsterdam) ^b^ | 88 (47%) | 71 (55%) | 101 (70%) |  | |
| Scan site (Nijmegen) ^b^ | 100 (53%) | 58 (45%) | 43 (30%) |  | |

Note, ^a^ Data are represented as mean ± standard deviation. ^b^ Data are denoted as a number (percentage).

**TABLE S2,** Subgroups partitioned by ages of 317 adolescents from ADHD families in the replication sample.

|  | Age range, # | ADHD (#) | Unaffected siblings (#) | Females (#) | Males (#) |
| --- | --- | --- | --- | --- | --- |
| Partition 1 | 7-15, 152 | 96 | 56 | 81 | 71 |
|  | 15-17, 165 | 92 | 73 | 62 | 103 |
| Partition 2 | 7-14, 108 | 68 | 40 | 65 | 43 |
|  | 14-16, 91 | 46 | 45 | 35 | 56 |
|  | 16-17, 118 | 74 | 44 | 43 | 75 |

Note, # denotes the number of participants.

**Table S3**, GMV IC 3-SNP component association in 341 adults (discovery samples), 461 adolescents (replication samples), and 317 adolescents from ADHD families in the replication samples, respectively.

| GMV IC 3-SNP component association | 341 adults | 461 adolescents | 317 adolescents from ADHD families |
| --- | --- | --- | --- |
| *p* | 0.56 | 0.80 | 0.17 |
| $\eta_{p}^{2}$ | 3.24×10^-3^ | 2.37×10^-3^ | 0.10 |

Note, $\eta_{p}^{2}$ denotes partial eta squared.

**Table S4**, Summary of 4 top SNPs regulation effects on gene expression, with rsID, chromosome number (Chr), base pair (BP) position, reference (Ref) allele, *p* and beta values of the regulation effect as well as regulated genes listed.

| rsID | Chr | BP position | Ref allele | p value | Beta | Regulated gene |
| --- | --- | --- | --- | --- | --- | --- |
| rs61104616 | 5 | 88163771 | G | 4.17E-02 | -0.05 | LINC00461 |
| rs1116527 | 6 | 149302053 | T | 1.19E-03 | -0.10 | RP1-111D6.3 |
| rs2842198 | 1 | 43930738 | A | 1.43E-08 | 0.13 | TIE1 |
| rs1313237 | 14 | 60848224 | C | 5.47E-03 | 0.05 | PCNXL4 |

Note, p values listed were after FDR correction, the reference allele was matched between my data and that from PsychENCODE (the same for Table S5 and S6).

**Table S5**, Summary of 6 top SNPs showed significant isoQTL effect, with rsID, chromosome number (Chr), base pair (BP) position, reference (Ref) allele, *p* and beta values of the regulation effect, regulated transcripts and their types listed.

| rsID | Chr | BP position | Ref allele | p value | Beta | Regulated transcripts | Transcripts Type |
| --- | --- | --- | --- | --- | --- | --- | --- |
| rs373098 | 5 | 88007261 | C | 3.22E-04 | 0.19 | MEF2C-023 | protein_coding |
| rs2362108 | 5 | 88147771 | A | 1.77E-04 | -0.19 | MEF2C-023 | protein_coding |
| rs1285992 | 14 | 91520438 | A | 7.76E-04 | -0.19 | RPS6KA5-004 | protein_coding |
| rs590247 | 15 | 47647560 | C | 3.46E-06 | 0.21 | SLC24A5-003 | retained_intron |
| rs6047270 | 20 | 21122212 | T | 2.17E-40 | 0.56 | PLK1S1-007 | processed_transcript |
| rs1313237 | 14 | 60848224 | C | 1.95E-06 | -0.22 | DHRS7-001 | protein_coding |

**Table S6**, Summary of 3 top SNPs showed significant tQTL effect, with rsID, chromosome number (Chr), base pair (BP) position, reference (Ref) allele, *p* and beta values of the regulation effect, regulated transcripts and their types listed.

| rsID | Chr | BP position | Ref allele | p value | Beta | Regulated transcripts | Transcripts Type |
| --- | --- | --- | --- | --- | --- | --- | --- |
| rs6047270 | 20 | 21122212 | T | 3.56E-29 | 0.32 | PLK1S1-007 | processed_transcript |
| rs2842198 | 1 | 43930738 | A | 1.94E-04 | 0.07 | TMEM125-001 | protein_coding |
| rs1313237 | 14 | 60848224 | C | 2.04E-05 | -0.14 | DHRS7-001 | protein_coding |

**Table S7**, Summary of 10 top SNPs regulation effects on methylation, with rsID, chromosome number (Chr), base pair (BP) position, reference (Ref) allele, and effect on GMV (local data), as well as the regulated CPG site and regulation effect on DNA methylation (DNAm) listed.

| rsID | Chr | BP position | Local SNP and GMV data | | Jaffe et al. (mQTL summary) | | |
| --- | --- | --- | --- | --- | --- | --- | --- |
|  |  |  | Ref allele | Effect on GMV | CPG site | Annotation | Effect on DNAm |
| rs935832 | 8 | 1145844 | T | higher GMV | cg26999501 |  | lower DNAm |
| rs17669107 | 8 | 1152849 | C | higher GMV | cg26999501 |  | lower DNAm |
| rs1116527 | 6 | 149302053 | T | higher GMV | cg01163931 | UST | lower DNAm |
| rs2400169 | 5 | 144526632 | T | higher GMV | cg00055771 |  | higher DNAm |
| rs7902146 | 10 | 63801030 | C | higher GMV | cg06318796 | ARID5B | lower DNAm |
| rs6557063 | 5 | 92493318 | C | lower GMV | cg06025774 |  | lower DNAm |
| rs56144910 | 5 | 88198557 | A | lower GMV | cg18498987 | MEF2C | higher DNAm |
| rs11777844 | 8 | 1144023 | T | lower GMV | cg07699771 |  | lower DNAm |
| rs28452470 | 7 | 121957582 | A | lower GMV | cg12076551 |  | higher DNAm |
| rs2811908 | 9 | 86247287 | G | lower GMV | cg01266338 | C9orf103 | higher DNAm |

Note, the reference allele was matched between my data and that from Jaffe’s study[10].

**Reference**

1 von Rhein D, Mennes M, van Ewijk H, Groenman AP, Zwiers MP, Oosterlaan J, et al. The NeuroIMAGE study: a prospective phenotypic, cognitive, genetic and MRI study in children with attention-deficit/hyperactivity disorder. Design and descriptives. Eur Child Adoles Psy. 2015;24(3):265-81.

2 Onnink AMH, Zwiers MP, Hoogman M, Mostert JC, Kan CC, Buitelaar J, et al. Brain alterations in adult ADHD: Effects of gender, treatment and comorbid depression. Eur Neuropsychopharm. 2014;24(3):397-409.

3 Arias-Vasquez A, Groffen AJ, Spijker S, Ouwens KG, Klein M, Vojinovic D, et al. A Potential Role for the STXBP5-AS1 Gene in Adult ADHD Symptoms. Behav Genet. 2019;49(3):270-85.

4 JMR U. WAIS-III: Nederlandstalige bewerking: Wechsler Adult Intelligence Scale-Ed. 3:Afname en scoringshandleiding (WAIS-III 2000)*.* Swets Test Publishers: Lisse; 2000.

5 Esteban O, Birman D, Schaer M, Koyejo OO, Poldrack RA, Gorgolewski KJ. MRIQC: Advancing the automatic prediction of image quality in MRI from unseen sites. PLoS One. 2017;12(9):e0184661.

6 Duan K, Chen J, Calhoun VD, Lin D, Jiang W, Franke B, et al. Neural correlates of cognitive function and symptoms in attention-deficit/hyperactivity disorder in adults. Neuroimage Clin. 2018;19:374-83.

7 Duan K, Jiang W, Rootes-Murdy K, Schoenmacker GH, Arias-Vasquez A, Buitelaar JK, et al. Gray matter networks associated with attention and working memory deficit in ADHD across adolescence and adulthood. Transl Psychiatry. 2021;11(1):184.

8 Strang G. Computational science and engineering*.* Wellesley-Cambridge Press; 2007.

9 Duan KK, Silva RF, Chen JY, Lin DD, Calhoun VD, Liu JY. Sparse Infomax Based on Hoyer Projection and Its Application to Simulated Structural Mri and Snp Data. I S Biomed Imaging. 2019:418-21.

10 Jaffe AE, Gao Y, Deep-Soboslay A, Tao R, Hyde TM, Weinberger DR, et al. Mapping DNA methylation across development, genotype and schizophrenia in the human frontal cortex. Nat Neurosci. 2016;19(1):40-7.

11 Liu J, Duan K, Jiang W, Rootes-Murdy K, Schoenmacker G, Buitelaar JK, et al. Gray matter networks associated with cognitive deficit in ADHD across adolescence and adulthood. medRxiv. 2020:2020.04.22.20059808.

12 Johnson MB, Kawasawa YI, Mason CE, Krsnik Z, Coppola G, Bogdanovic D, et al. Functional and Evolutionary Insights into Human Brain Development through Global Transcriptome Analysis. Neuron. 2009;62(4):494-509.

13 Kang HJ, Kawasawa YI, Cheng F, Zhu Y, Xu XM, Li MF, et al. Spatio-temporal transcriptome of the human brain. Nature. 2011;478(7370):483-89.

14 Colantuoni C, Lipska BK, Ye TZ, Hyde TM, Tao R, Leek JT, et al. Temporal dynamics and genetic control of transcription in the human prefrontal cortex. Nature. 2011;478(7370):519-U117.
